# Achieving universal health coverage in low- and middle-income countries through digital antimicrobial stewardship

**DOI:** 10.3389/fdgth.2023.1298861

**Published:** 2023-12-15

**Authors:** Idemudia Imonikhe Otaigbe

**Affiliations:** Department of Medical Microbiology, School of Basic Clinical Sciences, Benjamin Carson (Snr) College of Health & Medical Sciences, Babcock University/Babcock University Teaching Hospital, Ilishan Remo, Ogun State, Nigeria

**Keywords:** government commitment to tackle AMR, digital antimicrobial stewardship, digital health, antimicrobial resistance, universal health care

## Introduction

One of the greatest achievements in modern medicine has been the discovery of antibiotics (1). Antibiotics have proven to be very useful agents in the fight against infectious diseases as they have contributed immensely to a reduction in morbidity and mortality and a substantial increase in life-span (1). However, the inappropriate use of antibiotics has become a global problem (2, 3). The inappropriate use of antibiotics is associated with the emergence of antimicrobial resistance (AMR), increased length of hospital stay, increased mortality, and increased healthcare costs borne by the patient and health systems (4, 5). Specifically, AMR is a global challenge with potential adverse impacts on development, health systems and food security (6). The devastating impacts of AMR are worse in Low- and middle-income countries (LMICs) with fragile health systems, weak regulatory frameworks, and a paucity of data to drive effective public health decision making (7). A failure to curb AMR could result in about 10 million global deaths per year and 4.1 million deaths in Africa by 2050 (8, 9). Also by 2050 it is estimated that about 28.3 million people will be living in extreme poverty on account of increased costs of care due to rising rates of AMR (10). This would lead to increased social inequality and marginalization of vulnerable groups such as women, refugees, the poor and the illiterate (11). Other predicted adverse economic impacts of AMR by 2050 include a reduction in global Gross Domestic Product by about 2%–3.5%, shortfalls in economic output valued at about 100 trillion US dollars (US$), and a failure to achieve Universal Health Coverage (UHC) and the Sustainable Development Goals (SDGs), particularly in LMICs, with poorly funded fragile health systems (6, 10).

In response to the global security threat posed by AMR, the World Health Organization (WHO) in May 2015 adopted the Global Action Plan on AMR (12). A key objective of the Global Action Plan is “to optimize the use of antimicrobial medicines in human and animal health” (12). Member states are expected to achieve this objective by developing and implementing comprehensive National Action Plans (NAPs) on AMR which incorporate antimicrobial stewardship (AMS) programs (12). An AMS program is defined as “an organizational or system-wide health-care strategy to promote appropriate use of antimicrobials through the implementation of evidence-based interventions” (13). In this regard, the World Health Organization (WHO) has recommended AMS programs as an effective intervention for curbing inappropriate antibiotic use and AMR in healthcare facilities in LMICs (13).

The WHO is also spearheading Sustainable Development Goal (SDG) 3 which seeks to “ensure healthy lives and promote well-being for all at all ages” (14). SDG 3 includes fighting communicable diseases, achieving Universal Health coverage (UHC), financial risk protection, access to quality essential health-care services and access to safe, effective, quality, and affordable essential medicines and vaccines for all (15). However, efforts to curb AMR and achieve UHC and other goal targets of SDG 3 cannot occur in isolation as there is a clear association between effective AMS programs and the attainment of UHC in LMICs (16–19). Similarly, the adverse social and economic impacts of AMR could make it difficult for LMIC governments with fragile health systems to ensure sustainable financing of universal health coverage (16–19). Sadly, the implementation of AMS programs in LMICs is quite low due to a paucity of funds, lack of political will and lack of technical expertise (20, 21). Ineffective or inexistent AMS programs result in rising AMR rates, poor quality of health care delivery and ultimately an inability to achieve UHC (13). In contrast, the presence of effective AMS programs would help to reduce AMR rates, ensure equitable and sustainable access to effective antimicrobials and improve treatment outcomes from infectious diseases (13). Also, the integration of effective AMS programs with other health system components (i.e., adequate diagnostic microbiology services, efficient governance frameworks, effective Infection Prevention and Control protocols etc.) results in strengthening of health systems and the advancement of UHC (13). Therefore, efforts to achieve UHC in LMICs should go alongside scaling up AMS programs and addressing AMR by prioritizing investments for interventions with high impact, scalability, sustainability, and low complexity (11, 22). An example of such investments is investing in the inclusion of digital tools in AMS programs i.e., Digital Antimicrobial Stewardship DAS programs.

Clearly digital tools (e.g., electronic Health and Medical records, apps, telemedicine, electronic surveillance systems, health analytics, artificial intelligence etc.) can be adapted to scale up the implementation of AMS programs in LMICs and subsequently UHC as it has become increasingly clear that the battle against AMR and efforts to attain UHC both require the support of digitalization (23–30). Also a clear lesson that has emanated from the COVID 19 pandemic is that the integration of digital tools in healthcare delivery in LMICs and resource constrained settings can expedite public health decision making and considerably improve health outcomes (31).

In the discussion below are some emerging opportunities which available digital tools or platforms present for the implementation of DAS programs in LMICs and the impact of DAS programs on achieving UHC. In addition, possible challenges that lie ahead in the implementation of digital antimicrobial stewardship in LMICs are mentioned. Finally, some recommendations to surmount these challenges are briefly discussed.

## Emerging opportunities

In this article Digital Antimicrobial Stewardship (DAS) is defined by the author as the application of digital tools to enhance the implementation and outcomes of antimicrobial stewardship (AMS) programs (author's definition).

Several international guidelines recommend the inclusion of digital interventions within AMS programs (13, 23, 32, 33). Also, Frenkel et al., state that the best method to implement an effective AMS program is by incorporating advanced algorithms, artificial intelligence, machine learning and decision-making software into AMS programs (34). While these may be cost intensive in several LMICs, digital innovation such as mobile apps are cheap, low hanging fruits which can reduce the need for extra manpower, provide easy access to guidelines (and educational resources), improve prescribing practices and patient outcomes (34–37).

Digitalization can also enhance AMS processes and help overcome challenges associated with implementing these processes (23). For example, clinical decision support systems (CDSS) can aid physicians in correctly diagnosing bacterial infections and guiding the choice of empiric antibiotic therapy (37–40). By combining a patient's bio-data, clinical history and diagnostic results (i.e., laboratory and radiological) CDSS can facilitate improved clinical decision-making, optimized antimicrobial therapy and better treatment outcomes (37–40).

Another example of an AMS process, which can benefit from digitalization is prospective audit and feedback. Prospective audit and feedback is a key AMS process which is associated with a lot of paperwork, difficulty in identifying patients receiving inappropriate antibiotic therapy and difficulty communicating recommendations to prescribers (24, 25, 32). However these challenges can be overcome with digital platforms (e.g., computerized provider order entry systems or electronic medical records) which would allow an AMS team to easily identify patients on an antimicrobial agent and enter notes into the system to document and communicate recommendations to prescribers (23).

The outcomes of AMS programs can also be improved by digitalization (23, 24, 33, 41). Outcomes include: decreased antimicrobial consumption; increased antimicrobial appropriateness; reduced length of hospitalization; reduced mortality; reduced rates of antimicrobial resistant pathogens; and decreased costs of hospitalization (23, 24). For example, a study by Bond et al., showed that a CDSS was associated with changes in targeted antimicrobial use, decreased antimicrobial costs and decreased health care associated *Clostridium difficile* rates (41).

In addition, digitalization can serve to scale up AMS programs in LMICs (42, 43). For example mobile apps or online digital tools could help policy/decision makers in different regions of the world to easily adapt AMS tools and processes to suit the context and peculiarities of their region(s) (44, 45).

## Challenges with implementing digital antimicrobial stewardship in LMICs

Digital Antimicrobial Stewardship (DAS) can facilitate the successful implementation of AMS programs in LMICs, reduce AMR rates and ultimately lead to better health outcomes and the attainment of UHC (23–30). However, there are challenges regarding its implementation in LMICs (46–49).

A major challenge is a lack of political will by LMIC governments to strengthen health systems, achieve UHC, implement NAPs on AMR and embrace digital health (46–50). Political will refers to “the commitment of political leaders and bureaucrats to undertake actions to achieve a set of objectives and to sustain the costs of those actions over time” (51). Poor political will results in poor governance structures and impedes an enabling environment for private sector investment in DAS and other digital innovations which could play the dual role of curbing AMR and achieving UHC (48, 50, 52).

Closely related to poor political will is the poor adoption of digital health innovations by key stakeholders (e.g., health workers, leadership of healthcare facilities, health policy makers etc.) in LMIC health sectors for several reasons: poor infrastructure (e.g., lack of internet access, unreliable power supply, etc.) to support the adoption of digital health innovations; poor knowledge/ literacy of information, communication and digital technology among some health workers; organizational or healthcare facility barriers (e.g., inadequate funds to adopt digital innovations, lack of support or buy-in of the healthcare facility's leadership etc.); and the perception of health workers to digital innovations (e.g., some health workers may have the perception that digital health innovations are complex, time consuming and increase the burden of work) (46, 47, 53–60).

Another challenge is that health workers and other end users of digital health technologies in LMICs are often excluded from deliberations regarding the design and development of digital health innovations (58). As a result they (i.e., health workers and end-users of digital health technologies) are unable to make useful inputs and contributions which would ensure such digital health technologies are context specific and meet end-users' needs (58).

Difficulties with interoperability of digital health innovations may also hinder the adoption of DAS platforms, especially in healthcare facilities with existing digital health technologies. Interoperability refers to “the ability of two or more computer systems or pieces of software to exchange and subsequently make use of data” (60). Interoperable digital health technologies facilitate adoption as they build on existing technologies and enable health workers (with prior knowledge and technical expertise of existing digital health technologies) to easily adopt new digital health technologies (43, 61).

However, the deployment of digital health technologies in LMICs generates ethical concerns such as informed consent, data privacy, data sharing, accountability (i.e., who is accountable when digital health technologies fail) diversity (i.e., the socio-cultural diversities and contexts of several LMICs may not be suited to digital technologies designed in high income nations) and a dearth of strong governance or regulatory frameworks to address these (and other) ethical concerns (62–66). Finally, several digital health platforms in LMICs are deployed as short term stand-alone projects and are not integrated into the health systems of the host LMIC (67). Therefore such projects lack sustainability and fail to strengthen the nation's health system (67).

## Recommendations

Improved political will and commitment are required by LMIC governments to address AMR and achieve UHC (16, 43, 48). The agenda of LMIC governments must emphasize that achieving UHC and curbing AMR must occur together (16, 43, 48). Concerted efforts must occur in LMICs to scale up and integrate DAS programs and other relevant digital health initiatives into National Action Plans (NAPS) on AMR (68). Also, the Global Strategy on Digital Health (2020–2025) provides country level guidance on digital interventions for health system strengthening (69). The strategy emphasizes that digital interventions must be integrated into existing national health systems and that technical, financial and infrastructural support should be provided to LMICs to enable adoption of digital health (69).

Efforts should also be made by LMIC governments to remove infrastructural, social, economic, cultural and other barriers which could hinder the adoption or scaling up of DAS and its (i.e., DAS) potential impact on achieving UHC (29, 43, 70). Similarly, a multi-disciplinary approach (involving government, Artificial Intelligence or Digital Health companies, end-users of DAS platforms such as clinicians or pharmacists, and key stakeholders in the country's AMR/AMS landscape) is required to overcome implementation hurdles (e.g., infrastructural deficits, costs of acquiring AI systems, the digital literacy of end-users etc.) associated with the adoption of digital health technologies in LMICs (61). For example, LMIC governments will need to collaborate with digital health providers and donor agencies to provide training and capacity building for health workers regarding the adoption of digital health tools such as DAS platforms (61). In addition, anticipated end-users should be involved very early in the development of DAS platforms as this will allow the developers of the technology to receive important feedback from end users and tailor the DAS platforms to fit the needs and contexts of the end-users (58, 71). To ensure interoperability and achieve scale and impact, developers of DAS platforms should ensure that these platforms are built on existing digital health technologies (61). LMIC governments should also develop strong governance structures to address ethical concerns (e.g., data security and privacy) associated with the deployment of digital technologies (29, 62).

The private sector has a role to play in deploying DAS programs to curb AMR and achieve UHC in LMICs (29, 61). Similarly, the COVID-19 pandemic has accelerated the adoption of digital health technologies, globally, resulting in immense opportunities for private sector participation (61, 72, 73). However, in several LMICs, the private sector (and governments) lack the capacity to deploy digital health solutions on their own (61). Strong partnerships between LMIC governments and the private sector are therefore necessary to scale up the adoption of DAS programs (61) and LMIC governments should therefore create a conducive investment climate for private sector participation in efforts to implement DAS and achieve UHC (61). Private sector participation should however not adopt a “one size fits all” approach (74). Unique investment models (e.g., Public Private Partnerships; Joint Ventures etc.) will be required (75–77). These investment models will take into consideration the socio-cultural, economic and political dynamics of each LMIC (74–76). For example, Public Private Partnerships have been instrumental in expanding digital health services to rural parts of Africa, Asia and South America (78–80). However, the efforts of the private sector should be carefully regulated by LMIC governments to ensure digital health solutions are sustainable, integrated into NAPs on AMR and strengthen the nation's health system (81, 82). In addition, LMIC governments should ensure strong monitoring and evaluation frameworks are in place to ensure DAS platforms achieve expected outcomes (83).

## Conclusion

Tackling AMR and achieving UHC are closely linked global health priorities (48). A failure to curb AMR will impede the achievement of UHC (particularly in LMICs with weak health systems, high burdens of infectious diseases and high morbidity and mortality from infectious diseases) as health care delivery will become less effective, and more expensive (11, 48). Therefore, efforts to curb AMR must occur simultaneously with efforts to achieve UHC (11, 48). The introduction of Digital antimicrobial stewardship (DAS) programs in LMICs will optimize antibiotic use, improve treatment outcomes from infectious diseases, strengthen health systems and advance the achievement of UHC (11, 23, 24, 37, 48). However, LMIC governments should exhibit the required political will to successfully implement DAS programs (11, 48). In addition, the implementation of DAS is not the sole responsibility of the government; the private sector also has a useful role to play in the implementation of DAS programs in LMICs (29, 61, 72, 73). Therefore, LMIC governments should create a conducive investment climate for private sector participation and ensure strong governance frameworks are in place to guarantee sustainability, quality service delivery and strengthening of the nation's health system (61, 81–83).
